# Neural networks mediating sentence reading in the deaf

**DOI:** 10.3389/fnhum.2014.00394

**Published:** 2014-06-10

**Authors:** Elizabeth A. Hirshorn, Matthew W. G. Dye, Peter C. Hauser, Ted R. Supalla, Daphne Bavelier

**Affiliations:** ^1^Department of Brain and Cognitive Sciences, University of Rochester, Rochester, NY, USA; ^2^Learning Research and Development Center, University of Pittsburgh, Pittsburgh, PA, USA; ^3^Department of Speech and Hearing Science, University of Illinois at Urbana-Champaign, Champaign, IL, USA; ^4^Beckman Institute for Advanced Science and Technology, University of Illinois at Urbana-Champaign, Urbana, IL, USA; ^5^Department of Psychology, University of Illinois at Urbana-Champaign, Champaign, IL, USA; ^6^National Technical Institute for the Deaf, Rochester Institute of Technology, Rochester, NY, USA; ^7^Faculté de Psychologie et des Sciences de l'éducation, Université de GenèveGeneva, Switzerland

**Keywords:** reading, deaf, native signers, oral training, sentence comprehension, Superior Temporal Gyrus

## Abstract

The present work addresses the neural bases of sentence reading in deaf populations. To better understand the relative role of deafness and spoken language knowledge in shaping the neural networks that mediate sentence reading, three populations with different degrees of English knowledge and depth of hearing loss were included—deaf signers, oral deaf and hearing individuals. The three groups were matched for reading comprehension and scanned while reading sentences. A similar neural network of left perisylvian areas was observed, supporting the view of a shared network of areas for reading despite differences in hearing and English knowledge. However, differences were observed, in particular in the auditory cortex, with deaf signers and oral deaf showing greatest bilateral superior temporal gyrus (STG) recruitment as compared to hearing individuals. Importantly, within deaf individuals, the same STG area in the left hemisphere showed greater recruitment as hearing loss increased. To further understand the functional role of such auditory cortex re-organization after deafness, connectivity analyses were performed from the STG regions identified above. Connectivity from the left STG toward areas typically associated with semantic processing (BA45 and thalami) was greater in deaf signers and in oral deaf as compared to hearing. In contrast, connectivity from left STG toward areas identified with speech-based processing was greater in hearing and in oral deaf as compared to deaf signers. These results support the growing literature indicating recruitment of auditory areas after congenital deafness for visually-mediated language functions, and establish that both auditory deprivation and language experience shape its functional reorganization. Implications for differential reliance on semantic vs. phonological pathways during reading in the three groups is discussed.

## Introduction

Achieving literacy requires extracting meaning from text, requiring a series of intermediate steps. In alphabetic languages, it is well described that the first steps include becoming aware that words are made up of smaller units of speech sounds (phonological awareness), allowing the learner the insight required to link visual and phonological information as written words are decoded. For a typically developing reader, the decoded written words can then be mapped onto an established spoken word lexicon. Such decoding, however, presents a specific challenge for deaf individuals whose knowledge of the spoken language phonology is weaker. In addition, successful reading goes well beyond such decoding, as it also entails comprehension. Although often thought of as a byproduct of knowing a spoken language, *reading comprehension* is a second, yet arguably the most important, step of literacy. It requires a specific set of skills, including the command of the syntax of the language being read, and the ability to combine facts and concepts into coherent schemas (Cain et al., 2004).

Studying reading in deaf individuals is of particular interest because many aspects of learning to read that are often taken for granted are not applicable. One general assumption is that people know and speak the language that they learn to read. This assumption carries many implications, including that learners are familiar with the sounds, lexicon, and grammar of the language they learn to read. In profoundly deaf individuals, many of those assumptions are unlikely to be qualified, or even false. This has direct consequences for the type of cognitive problems a deaf reader needs to solve when acquiring literacy, the information useful in solving those problems, and the neural circuitry likely to be involved. The goal of the current study is to further our understanding of the neural basis of sentence reading and its underlying mechanisms in deaf individuals. In particular we aim to characterize the type of neuroplastic changes that may emerge in the face of such large differences in hearing and language experience. To address that aim, the present study includes three distinct populations. On the one hand, deaf native signers, individuals born within the Deaf community who are severely to profoundly deaf and early users of a signed language, and on the other hand, hearing individuals with normal hearing and no knowledge of signed language. As an intermediate population, oral deaf participants were included who had the criteria of being born with severe to profound hearing loss. Unlike deaf signers, this latter group uses oral language daily, and unlike hearing individuals, these individuals have hearing impairments.

### Reading and deafness

The reading literature, mostly guided by the study of hearing individuals, converges to suggest that speech-based phonological awareness is a key building block for achieving proficiency in reading comprehension and is needed to engage typical skilled reading networks (Wagner and Torgesen, 1987; Pugh et al., 2000; Shaywitz et al., 2002). Especially in an alphabetic script like English, decoding skills, or the ability to “sound words out,” is thought of as the foundation of reading, without which one would be impaired at single word reading and sentence comprehension (Snowling, 1998). Not surprisingly then, learning to read *is* difficult for deaf individuals as deafness is typically associated with weaker speech-based phonological skills. On average, deaf readers achieve only a fourth grade reading level (Traxler, 2000). The case of deaf individuals raises the issue of whether it is possible to successfully read when foundational skills like “sounding out words” are challenged.

The emphasis on phonological skills in the reading literature is echoed in research on reading in deaf population. Much of this research has focused on whether deaf individuals can attain speech-based phonological awareness and whether they use it in the service of reading (Harris and Beech, 1998; Nielsen and Luetke-Stahlman, 2002; Harris and Moreno, 2006; Kyle and Harris, 2006, 2010; Mohammed et al., 2006; Colin et al., 2007). This literature provides mixed answers, probably owing to the fact that the deaf population is extremely heterogeneous in regards to their level of dB loss, their language experience (having access to language through sign language, oral speech training, Total Communication, Exact Signed English, Cued Speech, and so on), and their age of language acquisition. Each of these factors will affect speech-based phonological awareness. For example, speech-based phonological awareness varies with language background such that those deaf individuals with experience with spoken language (oral training or cued speech) perform better than deaf signers (Koo et al., 2008). Interestingly, congenitally deaf individuals who primarily use sign language still demonstrate some level of phonological knowledge (Hanson and Fowler, 1987; Miller, 1997; Harris and Beech, 1998; Harris and Moreno, 2006; MacSweeney et al., 2008). Nevertheless, there is still much controversy concerning the nature of speech-based phonological knowledge in deaf individuals, especially those native signers who rarely or never use speech to communicate. The qualitative nature of phonological awareness (McQuarrie and Parrila, 2009) and whether such knowledge is *useful* when reading (Mayberry et al., 2011) continues to be studied in deaf individuals. For example, recent evidence suggests that while deaf readers activate orthographic representations while reading, they do not activate phonological representations based upon orthographic information available in parafoveal vision in the same way as hearing readers do (Bélanger et al., 2013), suggesting that deaf readers make less use of phonological information while reading.

As mentioned above, reading comprehension builds not only upon phonological skills but also upon syntactic and semantic knowledge. The role of syntax and semantics in literacy is highlighted by work on deaf individuals who are native users of American Sign Language (ASL) (Goldin Meadow and Mayberry, 2001; Koo et al., 2008). These are individuals who were born deaf to deaf parents, who do not use much spoken English if any, and were exposed from birth to a visuo-manual language such as ASL. Several works point to the facilitating role of being a native signer in achieving literacy in deaf populations (Traxler et al., 2014). Early access to a natural language is hypothesized to provide the necessary linguistic and metalinguistic knowledge needed to better master the challenges of English literacy acquisition (Chamberlain and Mayberry, 2000). The role of metalinguistic processes is also reflected in studies showing that deaf readers may rely more on top–down conceptual information during reading than do hearing readers (Kelly, 1995; Musselman, 2000; Perfetti and Sandak, 2000). Our recent research has more specifically suggested that deaf native signers rely on a different reading style, such that their reading comprehension is more highly correlated with a general memory measure linked to semantic processing, whereas comprehension in hearing individuals is more strongly correlated with speech-based phonological knowledge (Hirshorn, 2011). Interestingly, deaf individuals who had been orally trained were found to show significant reliance on speech-based phonological knowledge compared to deaf native signers, highlighting the importance of language background in promoting routes to literacy in deaf populations. The current study includes both deaf native signers and oral deaf in order to test whether there is evidence for differential reliance on phonological and semantic processing in the neural signature of reading in these two groups.

### Neural networks for reading

Studies of literacy in hearing individuals have identified major contributions of both *phonological processes* as the orthographic input is decoded into sound, and of *semantics* as meaning is retrieved (Plaut et al., 1996; Coltheart et al., 2001). In terms of neural processing, orthographic information first enters the visual cortex and is processed in the much-debated inferior temporo-occipital cortex (Price and Devlin, 2003; Devlin et al., 2006), often referred to as the visual word form area (VWFA) (Cohen et al., 2002; Cohen and Dehaene, 2004). From there, the literature describes two left-lateralized networks that mediate literacy (Fiebach et al., 2002; Jobard et al., 2003; Turkeltaub et al., 2003; Borowsky et al., 2006; Cohen et al., 2008; Dehaene, 2009). The dorsal reading network, thought to underlie the linking of orthographic representations to their phonological codes, includes superior temporal areas, the supramarginal gyrus, the pre- and post-central gyri, and the opercular part of the inferior frontal gyrus (BA44). In contrast, the ventral reading network, thought to underlie lexically-based semantic processing of words, includes the middle temporal gyrus and anterior temporal cortex and the LIFG pars triangularis (BA 45/47) (Glasser and Rilling, 2008). Interestingly, these two reading networks mirror and overlap speech processing networks (Hickok and Poeppel, 2000; Saur et al., 2008). The dorsal speech network is similarly thought to be involved in processing phonological speech signals, which are then used for sensory-motor planning and speech articulation. In contrast, the ventral speech network is thought to mediate the processing of speech signals for comprehension. Taken together, there is overwhelming evidence that the dorsal neural network areas are biased toward phonologically-related processing, whereas the ventral neural network areas are biased toward semantically-related processing (Pugh et al., 1996; Sandak et al., 2004). However, as speech-based phonological experience becomes degraded by congenital deafness, it remains unclear how these respective reading networks would develop.

### Functional segregation in reading networks in deaf populations

Of special interest to our study is the functional distinction between sub-regions in the left inferior frontal gyrus (LIFG) whereby BA 45/47 underlies lexico-semantic processing and BA 44 shows greater association with phonological, or in the case of sentences also syntactic, processing (Dapretto and Bookheimer, 1999; Ni et al., 2000; Keller et al., 2001; Newman et al., 2001; Friederici et al., 2003). One goal of the current research is to determine the relative contribution of these dorsal and ventral networks during sentence reading in deaf individuals. In contrast to hearing individuals, congenitally deaf individuals likely have a degraded level of speech-based phonological knowledge, a weaker knowledge of spoken language syntax, and may rely to a greater extent on semantic/conceptual information. Thus the ventral network, including BA 45, may play an important role in reading processing for deaf readers, especially deaf native signers who are expected to have the weakest phonological knowledge and greatest dB loss of the groups studied.

Previous studies have started to explore the neural networks involved in deaf reading, but mostly at the single word-level (Aparicio et al., 2007; Waters et al., 2007; Corina et al., 2013; Emmorey et al., 2013). During tasks that required speech-based phonological knowledge, Emmorey et al. (2013) and Aparicio et al. (2007) both observed greater activation in deaf readers than hearing individuals within regions of the dorsal network, particularly within the left parietal cortex and bilateral frontal cortex. These results are also supported by greater left inferior frontal activation in deaf signers compared to hearing observed in a rhyme-judgment task (MacSweeney et al., 2009) and a phonological working memory task (Rudner et al., 2013). Emmorey et al. (2013) additionally reported a greater functional segregation within the LIFG in deaf than in hearing readers between anterior BA 45/47 (for semantic processing) and posterior BA 44 (for phonological processing). One possible explanation for this greater segregation rests on the idea that deaf readers may rely more on direct orthographic-to-semantic mapping, and thus less likely to implicitly activate phonological representations upon reading a word, compared to hearing readers who automatically activate phonological representations (Perfetti et al., 1988). Therefore, deaf readers may not have an inherent link between phonological and semantic representations in the same way as hearing readers do. Less is known about the functional status of subparts of the LIFG during sentence reading. The only available studies of sentence reading in the deaf are by Neville et al. (1998) and Newman et al. (2002), and both contrasted sentence reading and ASL processing in deaf native signers and hearing individuals. They found very similar fronto-temporal activity in all groups. However, because the analyses grouped BA 44 and 45 together, they leave the contribution of each pathway to sentence reading unspecified. Of note, across all available studies that compared deaf and hearing readers, the overall networks involved were largely similar and encompassed left perisylvian cortex and inferior frontal areas. This suggests that similarly organized ventral and dorsal networks also mediate reading in the deaf.

### Impact of cross-modal reorganization within the auditory cortex on reading networks in the deaf

The literature on deaf individuals, in particular deaf individuals with early profound deafness such as those included here, has documented the potential for the auditory cortex at large to undergo cross-modal reorganization. In this view, the auditory cortex deprived of its stereotyped source of sensory stimulation may become coopted by other modalities. While there is much debate as to the capacity of primary auditory cortex to undergo cross-modal reorganization (Finney et al., 2001; Kral et al., 2003; Karns et al., 2012; Scott et al., 2014), there is converging evidence that as a result of sensory deprivation, the auditory cortex at large reorganizes to support some aspects of non-auditory processing (Bavelier and Neville, 2002; Bavelier et al., 2006; Kral, 2007; Merabet and Pascual-Leone, 2010; Frasnelli et al., 2011). The existing literature, for example, documents the recruitment of auditory areas in congenitally deaf individuals during signed language processing (Nishimura et al., 1999; MacSweeney et al., 2002, 2006; Cardin et al., 2013). The paper by Cardin et al. (2013) is of particular interest here, as they attempted to separate the effects of sign language experience from those of auditory deprivation. Like in the current study, they compared deaf native signers, hearing, and additionally oral deaf who have similar sensory experience as deaf native signers, but similar language background as hearing individuals. In a study where participants were viewing British or Swedish Sign Language, they reported an effect of sign language experience in the left STG, in contrast to an effect of auditory deprivation in the right STG. This corresponds well with previous studies that have examined recruitment of classic auditory structures for processing non-linguistic visual inputs that have also reported a right lateralized recruitment of the STG (Finney et al., 2001, 2003; Fine et al., 2005; Vachon et al., 2013).

While the underlying mechanisms that support such complex non-typical brain specialization after deprivation are not yet entirely elucidated, an emerging view is that cross-modal reorganization in the deaf is most likely to occur in areas that are involved in supra-modal computations (Bavelier and Hirshorn, 2010; Lomber et al., 2010). Supra-modal computations are those functions that are not specific to one modality, but rather may be mediated by several modalities such as object localization or the processing of the direction of motion (e.g., both visual and auditory processing can mediate object localization or motion processing). As auditory areas are typically recruited for mediating language processing functions in speech, they may come to mediate similar language processing functions in the deaf, but now for visually-mediated language such as ASL or reading. To date, there is little evidence for auditory structures in the deaf being recruited for written language processing. One aim of the present study is to evaluate the participation of core auditory areas to sentence reading in the deaf populations studied, and evaluate the possible roles of dB loss and English phonological knowledge in such cross-modal reorganization if observed. Additionally, if the auditory cortex is found to undergo cross-modal plastic changes, we will further document the functional role of such reorganization by probing how the reorganized auditory cortex connects to the ventral and dorsal reading networks.

### Current study

By focusing on sentence reading of academically successful deaf readers, the current study seeks to ask about the networks that mediate literacy in profoundly deaf individuals as a function of their language experience and level of spoken English ability. Deaf native signers, oral deaf, and a hearing baseline group, all within a comparable range of reading ability were compared. The deaf native signers and oral deaf both had severe-to-profound hearing loss and access to a natural language early in childhood (ASL or English), but different levels of speech-based phonological knowledge. Oral deaf and hearing individuals shared knowledge of speech-based phonology, although that of oral deaf was likely to be less complete than that of the hearing, and the two groups differed markedly in terms of hearing loss. The inclusion of these three populations should therefore begin to inform us about the relative contribution of deafness and phonological knowledge (even if they do not provide a perfect orthogonal contrast of these factors). In addition, this study collected background information and behavioral data from tasks that have been shown to be reliable predictors of reading comprehension (IQ, speech-based phonological knowledge and free recall memory).

## Materials and methods

### Participants

All participants were treated in accordance with the University of Rochester's Research Subjects Review Board guidelines and were paid for their participation in the study.

#### Deaf native signers and oral deaf

Common inclusion criteria for all deaf participants included: (i) unaided dB loss of 75 dB or greater in the better ear^1^ (ii) onset of deafness before 2 years of age^2^. All participants were right-handed and none were fitted with a cochlear implant. All deaf participants were students at the Rochester Institute of Technology or the National Technical Institute for the Deaf and had been attending university for an average of 2.5 years (range = 0.5–6 years). No participants had any reported learning disorders.

There were 16 deaf participants [*M*_age_ = 22 years (range = 18–32); 9 female] who were native signers of ASL. Additional inclusion criteria for deaf native signers were (i) being born to at least one deaf parent, (ii) exposed to ASL from infancy, and (iii) using ASL as their mode of communication and with spoken language skills deemed being too poor to support communication. All deaf native signers reported having used hearing aids at some point in their lives, but only four continued to use hearing aids regularly for environmental sounds. Thirteen deaf native signers attended a school for the deaf during at least one phase of their education before college, and three attended a mainstream school throughout.

There were 12 oral deaf participants [*M*_age_ = 21 years (range = 18–24); 6 female] who had been trained to speak and lip-read English during their school years. In addition to the common criteria across the two deaf groups, specific inclusion criteria for oral deaf subjects were (i) being born to hearing parents, (ii) being educated in mainstream schools that adopted oral-aural approaches that promoted spoken language ability, (iii) minimal or absent ASL skills with no exposure to ASL until college years, (iv) using oral communication as the primary mode of communication, and relying on lip-reading to comprehend spoken English. All oral deaf participants except one reported using hearing aids for both environmental sounds and aiding for speech perception. Every oral deaf participant reported heavily relying on lipreading to comprehend speech. Typically such students received individual speech therapy on a regular basis upon entering the school system and continued to receive speech training as a part of all of their academic courses. They would have accessed information in the classroom through lip-reading their teachers. Four had attended a preschool for deaf children, but all attended mainstream schools during their elementary, middle and high school years. Six reported not using ASL at all, while the other six reported having developed some experience with ASL, but only starting in college.

Inclusion/exclusion criteria for language preference were assessed using a detailed background questionnaire about language use in school and at home, as well as checked through the fluency tests administered in English and in ASL [see section Non-verbal IQ (TONI-2) and Table 1].

**Table 1:** **Group characteristics**.

**Native language fluency accuracy**	**Deaf native signers**	**Oral deaf**	**Hearing**	***F***	***P***
English	N/A	0.32 (0.16)	0.66 (0.09)	54.31	<0.001
ASL	0.68 (0.12)	0.02 (0.04)	N/A	327.6	<0.001
Hearing loss (dB)	92 (10)	80 (13)	N/A	6.31	0.02
Non-verbal IQ (TONI-2)	98.9 (10.3)	98.3 (10.9)	93.6 (14.9)	0.79	0.46

Plotted are the group means for unaided dB loss, Non-verbal IQ standardized score, native language fluency accuracy (% correct), (standard deviation).

#### Hearing baseline group

There were 16 right-handed hearing participants [*M*_age_ = 24 years (range = 18–33); 7 female] with no hearing loss or experience with sign language or reports of learning disorders. They were recruited from the Rochester metropolitan area with the criteria that high school be the highest level of education completed (in order to match reading comprehension skills with the deaf groups). Hearing participants were either working or taking some college classes.

### Population descriptive statistics

#### Hearing level

We were unable to acquire the unaided dB loss for 1 oral deaf participant and 2 of the deaf native signing participants. Participants in both deaf groups were selected to have severe to profound hearing loss [mean dB loss in better ear: for deaf native signers = 92 (range = 70–105); for oral deaf = 80 (range = 65–98)], although a significant group difference was still observed (see Table 1). Hearing levels were obtained either from self-reports or consented/IRB approved access to RIT/NTID records.

#### Non-verbal IQ (TONI-2)

The TONI-2 was used as a test of non-verbal spatial intelligence (Brown et al., 1997). Participants viewed visual patterns with one missing component, of increasing complexity, and had to complete them with a multiple choice of 4 or 6 options. This test allowed us to control for the impact of general cognitive factors in reading comprehension. We note that, due to a communication error early during data collection, some participants were not given the TONI-2 and thus we are missing TONI-2 data for 3 deaf native signers. Data is reported as the standardized score (Mean = 100, *SD* = 15).

#### Language fluency

The American Sign Language Sentence Reproduction Test (ASL-SRT) was used as a test of ASL fluency (Hauser et al., 2008, under review). The Test of Adolescent Language Speaking Grammar Subtest (TOAL-2; Hammill, 1987) was used as a test of English fluency. In either test, participants were presented with videos of a model enunciating sentences of increasing length and complexity, either in ASL or in English. At the end of each sentence, participants were asked to repeat the sentence back precisely as was presented. Only sentences recalled verbatim were counted as correct. All participants in each group were asked to do both tasks, however deaf native signers were told that they could respond using ASL during the TOAL-2, in the case they did not feel comfortable vocalizing. Both hearing and oral deaf were instructed to watch the ASL sentences being signed and do their best to repeat back exactly what they saw. Hearing individuals were unable to correctly recall even the simplest ASL sentences, while oral deaf found the task extremely challenging. Two deaf native ASL signers scored the ASL fluency test (for deaf native signers and oral deaf subjects) with extremely high inter-subject reliability, *r*_(26)_ =0.97, *p* < 0.001, and a hearing native English speaker scored the English test (for hearing and oral deaf subjects). The percent accuracy (number of sentences repeated verbatim divided by the total) on each fluency test was compared between groups (see Table 1 for mean values).

### Behavioral determinants of reading

#### Reading comprehension

A test of English reading comprehension that did not require overt spoken responses was selected to evaluate reading skill, as it is unnatural for most deaf adults, especially native signers, to read aloud. All participants completed the Peabody Individual Achievement Test-Revised: Reading Comprehension (Markwardt, 1998), which is commonly used with deaf populations because it evaluates reading comprehension at the sentence level via nonverbal responses and has no speech production requirement (Morere, 2012, but for a critique see Keenan et al., 2008). Participants read sentences (with increasing length/number of clauses and less frequent vocabulary) one at a time and decided which of four pictures best matched each sentence. Non-matching pictures were foils designed to catch readers that were not carefully reading the text. Thus, a reader must completely understand the grammar and vocabulary of the sentence in order to select the correct picture match. Instead of focusing on decoding and pronunciation, as many reading tests do, this test focuses on lexical-semantics *and* syntactic knowledge of English.

The following cognitive factors that we have shown to be correlated with reading comprehension in these groups were measured (Hirshorn, 2011).

#### Speech-based phonology

Performance on a battery of tasks tapping different levels of speech–based knowledge was combined to provide an index of speech-based phonological knowledge that included tests of phonological knowledge at the phoneme, syllable, and rime levels, as well as speechreading (Hirshorn, 2011). Scores on each task were converted to z-scores and then averaged to create the combined speech-based phonological knowledge index. See Supplemental Information (SI) for a brief overview of each task and (Hirshorn, 2011) for a full description.

#### Free recall memory

Participants were presented with lists of 16 words and asked to recall in their preferred language as many words as possible in any order. Span was defined as the number of items recalled correctly (Rundus and Atkinson, 1970). All participants saw two lists, one in ASL and one in English. To avoid spurious list effects, each subject received a unique randomization of the 32 words, divided into two lists of 16 words. The lists were created by presenting the video of a model enunciating one word at a time either in English (audio-visual) or ASL (visual only) at a rate of 1 word every 5 s. Although participants saw one list in each language and were told to try their best, we only report performance in the preferred language as defined by the list on which they scored best. It is worth noting that each and every deaf native signer scored best when viewing an ASL list. In contrast, all oral deaf and hearing individuals scored best when viewing an English list.

### fMRI experiment methods

#### Stimuli and methods

Sentences were presented one word at a time in the middle of the screen (1 word/600 ms) in an event-related design, with a total 132 sentences distributed across four runs. In addition, “falsefont sentences” trials were presented in a similar fashion, one item/600 ms. These were created by converting real sentence stimuli to Wingdings font. Altogether 75% of the trials were real sentences (132) and 25% falsefont sentences (44). To promote reading for comprehension, twenty-five percent of the English sentences were followed by picture probes, which were drawn specifically for the experiment. Upon seeing the picture, the participant had to decide whether it matched or not the sentence they just read. Half of the picture trials matched the preceding sentence and half did not (see SI for more details). Trials in which the sentence was followed by a picture were coded separately and not analyzed. Importantly, participants could not predict if a picture probe would appear, in an effort to promote reading for comprehension throughout the experiment (in every run there could be either eight or nine picture probes; see SI for more details on task). Finally, participants were instructed to pay attention because there would be a post-test at the end of the scanning session where they would have to decide whether a given sentence or falsefont sentence had been presented during the scan.

All participants responded using button boxes attached to their feet. A foot response was preferred over manual response to avoid confounding between response mode and familiarity with sign language that may also lead to recruitment of the “hand” area in the somatosensory cortex. The stimulus presentation order with interleaved fixation (0–18 s) was determined using optseq2 (http://surfer.nmr.mgh.harvard.edu/optseq/).

#### Image acquisition for fMRI tasks

The experiment was carried out using a Siemens Trio 3T scanner at the Rochester Center for Brain Imaging, University of Rochester. We used a standard clinical quadrature radio-frequency head coil; foam padding was used to restrict head motion. A standard gradient-echo image acquisition using an echo planar pulse sequence was used to detect susceptibility-based (BOLD) contrast. Thirty contiguous oblique axial slices were obtained per acquisition, with flip angle 90°, 30 ms effective TE, a TR of 2 s, FOV 256 mm and a 64 × 64 matrix, resulting in a voxel size of 4 × 4 × 4 mm. Each imaging protocol started with an 8.5 min T1-weighted structural MRI (MPRAGE sequence), *TR* = 1960 ms, *TE* = 3.93 ms, 176 slices in a 256 × 256 matrix, voxel size 1 × 1 × 1 mm.

#### fMRI analysis

Images were converted to AFNI format from DICOM using an in-house script. Slice timing correction was performed (using Fourier interpolation in AFNI's 3dTshift program) to align the acquisition time of all slices to the same time point, as well as head movement correction using a rigid body (6-parameter) model in AFNI's 3dvolreg program. Images were spatially smoothed with a Gaussian kernel having a full width at half maximum of 2 times the voxel dimensions (8 mm), using AFNI's 3dmerge program. Statistical analyses were performed using AFNI's 3dDeconvolve software program. For each subject, a voxel-wise multiple regression was performed using a general linear model approach. The expected hemodynamic response to each word was modeled by a 1 s event convolved with a gamma function. The full regression model included motion parameters and modeled main effects of each condition (sentences and falsefont) and contrasts between conditions. Structural and functional normalization to standard space was done using *@auto_tlrc* and *adwarp* functions.

#### Group analyses and conjunction analysis

From the individual subject analyses, group-level activation maps were obtained for each of the conditions (sentences and falsefont contrasted with the fixation baseline) as well as for the relevant contrast (sentence vs. falsefont) using 3dANOVA2. All group-level analyses were thresholded at a corrected alpha value of *p* < 0.05, determined using Monte-Carlo simulations via AFNI's 3dClustSim program (cluster size of 15 voxels at *p* < 0.005). In preparation to look at group differences for the sentence vs. falsefont contrast, to ensure that only positive sentence activation was considered (that is, sentence activation > zero AND sentences > falsefont), we defined a mask as the union of all positive activation across all three groups for the contrast sentences vs. fixation. That union mask was applied to each of the group's sentences vs. falsefont contrast map, and the resulting masked map was used in the subsequent conjunction analysis and group comparisons. The most important check remains that % bold change in the areas of interest determined by group comparisons was seen to be greater for sentences than falsefonts in the groups with higher activation.

A conjunction analysis was performed on the contrast between sentences and falsefont to identify which areas within the reading networks were common to all three groups. The areas of activation for the contrast sentences vs. falsefont at *p* < 0.05 (corrected) for each group were overlaid to create a mask that included only the areas common to all three groups at that significance level (http://afni.nimh.nih.gov/sscc/gangc/ConjAna.html). Regions where each of the three groups showed significant positive activation for sentences vs. falsefont were defined as conjunction regions.

Finally, when considering group differences, AFNI's 3dttest program was used to examine the differences between deaf native signers vs. hearing for the contrast sentences vs. falsefont. The identified ROIs from the group comparison (i.e., bilateral STG and VWFA, see section Group Differences) were then used to extract % bold signal from each population allowing us to perform pair-wise comparison with oral deaf (i.e., hearing vs. oral deaf; deaf native signers vs. oral deaf).

#### Functional connectivity analyses

Due to lack of power from using a fast event-related design, we ran analyses on the time-series of the whole runs instead of using condition-specific psychophysiological interactions (O'Reilly et al., 2012). Seeding took place from the functionally defined STG ROIs from contrasting deaf native signers vs. hearing, and anatomically defined BA 44 and 45, based on sulci boundaries and the “TT_Daemon” Talairach-Tournoux anatomical atlas within the AFNI program that identifies the Brodmann areas of each voxel. The mean time series for all four runs was extracted for each subject for each individual ROI seed region. A simple functional correlation analysis was conducted using a standard procedure within AFNI (http://afni.nimh.nih.gov/sscc/gangc/SimCorrAna.html). Time series were used as a regressor in single-subject analysis using the standard GLM methods including nuisance variables that modeled head motion, white matter, CSF, and the global mean.

Group differences in functional connectivity from a given seed region (e.g., left STG, etc.) between deaf native signers and hearing readers were then computed using AFNI's 3dttest, and only results that were significant at the *p* < 0.05 corrected level were included—these defined significant projection regions. Among the significant projection regions, BA 45 and postcentral gyrus were of special interest given our initial aims. Connectivity strength from the STG to these significant project regions was then tabulated for each individual subject separately to run additional pairwise comparisons contrasting the oral deaf group to deaf native signers and to hearing individuals respectively (these analyses were carried outside of AFNI). Finally, given our interest in the functional segregation between BA 45 and BA 44 during language processing, each of these regions was anatomically defined (see above) and a connectivity analysis seeding from each was performed.

## Results

### Behavioral data

Levels of hearing loss (dB) were only compared in the two deaf groups, with oral deaf showing less severe hearing loss than deaf native signers. Importantly, the three groups did not differ in Non-verbal IQ (Table 1).

Because the hearing individuals were not able to perform the ASL Fluency task and deaf native signers were not able to perform the English Fluency Task, One-Way ANOVAs were run to compare ASL Fluency between deaf native signers and oral deaf, and English Fluency between hearing and oral deaf participants. Oral deaf showed intermediate performance between deaf native signers and hearing individuals, with deaf signers showing much higher ASL fluency and hearing showing higher English fluency (see Table 1 for One-Way ANOVA results on these measures).

The three groups did not significantly differ in reading comprehension scores (PIAT, grade equivalent level), *F*_(2, 41)_ = 2.07, *MSE* = 8.48, *p* = 0.14 (Table 2). As predicted, speech-based phonology skill (composite z-score, see SI) differed across groups such that the hearing group had the highest scores, followed by the oral deaf, and then deaf native signers. Free recall memory span also differed between groups, although past research indicates that deaf and hearing do not typically differ in this measure (Hanson, 1982).

**Table 2:** **Group performance on behavioral measures**.

	**Deaf native signers**	**Oral deaf**	**Hearing**	***F***	***p***
Reading comprehension (PIAT)	6.9 (3.36)	7.25 (2.51)	8.88 (2.69)	2.07	0.14
Speech-based Phonology	−0.72 (0.58)	0.38 (0.47)	0.41 (0.51)	22.78	<0.001
Free recall	8.47 (1.45)	9.17 (2.16)	11.18 (2.1)	8.33	0.001

Plotted are the group means for reading comprehension (PIAT) grad equivalent scores, speech-based phonological knowledge z-score index (see SI) and free recall memory span in the preferred language (ASL for deaf signers and English for oral deaf and hearing) (standard deviation).

### fMRI results

#### Behavioral results during fmri tasks

Performance on picture-trials during the scan was well above chance and did not differ across groups, hearing: *M* = 0.87 (±0.12), oral deaf: *M* = 0.87 (±0.13), deaf native signers: *M* = 0.89 (±0.11), *F*_(2, 41)_ = 0.119, *MSE* = 0.006, *p* = 0.88.

#### Reading networks as a function of group and conjunction analysis

For each group, the areas contributing to sentence reading were identified through the contrast sentences vs. falsefont (see SI Tables S1-S3). Overall these analyses identified in each population a rather standard network of areas around the left perisylvian region and its right homologous areas.

A conjunction analysis was then performed to confirm the areas common to all three groups. The areas found to be activated more for sentences than falsefont in all three groups include typical left perisylvian reading network areas such as the mid and superior temporal cortices extending to the left inferior temporal cortex and left inferior frontal cortex, in addition to the insula, pre-central and post-central gyri, and the right homologs of temporal and frontal regions (see Table 3 for all areas).

**Table 3:** **Reading network common to all three groups as determined by conjunction analysis**.

**Label**	***BA***	**No. Voxels**	***X***	***Y***	***Z***
**LEFT**					
Superior/Middle/Inferior temporal gyrus/Fusiform gyrus	22/21/20	303	−38	−29	−20
Inferior frontal gyrus/Insula	47/45/13	105	−50	27	−4
Pre-/Postcentral gyri	4/3	22	−50	9	44
**RIGHT**					
Insula/Inferior frontal gyrus	13/45/47	76	38	27	0
Middle temporal gyrus	22	56	50	21	−8

#### Group differences

**Figure 1:**
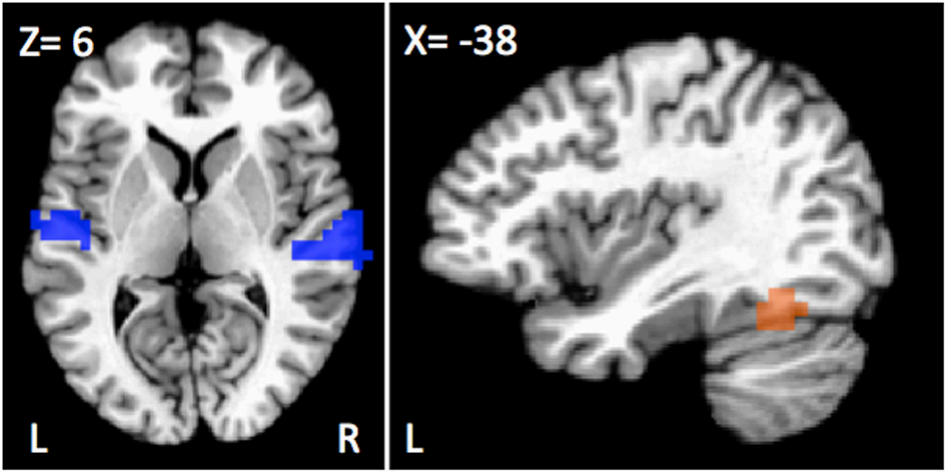
**Regions showing differences in activation as a function of population for the sentences vs. falsefont contrast**. Bilateral superior temporal cortices, including the left primary auditory cortex (blue) showed greater recruitment for deaf individuals, signers or oral, than hearing participants. Left fusiform (orange) showed a different pattern with greater activation for hearing and oral deaf participants as compared to deaf native signers.

A first analysis focused on differences between deaf native signers and hearing participants for the contrast of sentences vs. falsefont. Only two main regions showed group differences—a large portion of the bilateral STG and a region overlapping with the standard VWFA in the inferior temporal gyrus (see Figure 1).

Greater sentence activation was observed in deaf native signers than in hearing individuals in the superior temporal gyrus (STG) bilaterally, spanning from Heschl's Gyrus all the way to more posterior and ventral auditory cortex areas (Left STG; peak activation *X*, Y, Z = −62, −13, 8, 36 voxels; *t*_(30)_ = 4.00, *d* = 1.46, *p* < 0.001; Right STG; peak activation *X*, Y, Z = 66, −25, 12, 51 voxels; *t*_(30)_ = 4.65, *d* = 1.70, *p* < 0.001). Similar differences were observed when comparing oral deaf to hearing individuals such that oral deaf had greater bilateral STG activity than hearing individuals Left STG: *t*_(26)_ = 2.36, *d* = 0.93, *p* = 0.026; Right STG: *t*_(26)_ = 2.55, *d* = 1.00, *p* = 0.017). The oral deaf did not significantly differ from the deaf native signers (Left STG: *t*_(26)_ = 0.071, *d* = 0.09, *p* = 0.94*;* Right STG: *t*_(26)_ = 0.23, *d* = 0.02, *p* = 0.82).

Another group difference of interest was observed in the left fusiform gyrus that falls within the area often described as the visual word form area (VWFA; peak activation *X*, Y, Z=−42,−57, −16, 20 voxels) that showed greater activation for hearing than deaf native signers, [*t*_(30)_ = −4.13, *d*=−1.51, *p* < 0.001]. In this case, the oral deaf were not different from the hearing group [*t*_(26)_ = −0.43, *d* = −0.17, *p* = 0.67], but differed from deaf native signers [*t*_(26)_ = 2.69, *d* = 1.05, *p* = 0.012].

#### Understanding neuroplastic changes in the STG

Greater recruitment of the STG in deaf individuals led us to explore the relative contribution of language experience and dB loss to this reorganization. Regression analyses were conducted excluding the hearing group, as participants would be at floor on measures of dB loss and ASL fluency, and thus would skew the analyses. Of interest, was the level of STG recruitment as a function of dB loss, ASL fluency, and speech-based knowledge when considering together all deaf participants. Although a first regression analyses identified dB loss as the best predictor of left STG recruitment, *R*^2^= 0.20, *F*_(1, 26)_ = 5.74, *p* = 0.025, the interpretation of this result is weakened by the fact that dB loss, ASL fluency and English knowledge are somewhat correlated. To more precisely assess the unique contribution of each measure, residuals for each measure, regressing out any shared variance due to the other two measures, were first computed. A stepwise regression analysis was then performed using these three residual measures against STG recruitment. Using the left STG activation as the dependent measure, and as independent variables all three residuals, plus Nonverbal IQ, Reading Comprehension, and Free Recall, dB loss was the only significant predictor, *R*^2^ = 0.18, *F*_(1, 26)_ = 5.16, *p* = 0.033. In other words, after dB loss was accounted for, no other predictor significantly accounted for any of the remaining variance. Thus, greater dB loss was linked to greater activation in that left STG auditory cortex. In similar analyses that included only the two deaf groups, no measure correlated with right STG activity (all *p*s > 0.43 whether raw measures or residuals were used).

The previous analyses identified the left STG, and thus a large part of the auditory cortex, as a locus of cross-modal reorganization that correlated with dB loss in the deaf groups. To better understand the functional role of that reorganization, we carried an analysis of the functional connectivity of this area and in particular asked where group differences in functional connectivity from that area exist. Since our interest is in group differences in functional connectivity, we first report connectivity differences between the two extreme groups, deaf native signers and hearing participants (using a corrected *p* < 0.05 threshold). Seeding from the left STG, areas that showed greater connectivity in deaf native signers than hearing individuals included bilateral inferior frontal regions (including BA 45), anterior cingulate, angular/inferior/superior parietal cortex, right occipital cortex, and bilateral thalami. Conversely, areas that showed greater connectivity from left STG in hearing individuals included bilateral pre- and postcentral gyri and bilateral posterior insula (see Table 4 for full list). Similar results were found when seeding from right STG (see SI for full list).

**Table 4:** **Regions in which functional connectivity with left STG differed between hearing and deaf native signers, *p* < 0.05 (corrected)**.

**Label**	***BA***	**No. Voxels**	***X***	***Y***	***Z***
**DEAF NATIVE SIGNERS > HEARING**					
**Left**					
Insula	13	24	−34	19	4
Inferior frontal gyrus	45	71	−54	19	16
Angular gyrus/Precuneus	39	21	−30	−61	40
**Right**					
Superior frontal/Cingulate	6/32	130	6	35	56
Inferior/Middle frontal gyrus/Insula	47/9/13	135	42	23	−4
Bilateral thalamus		292	2	−21	16
Superior parietal lobule	7	63	38	−65	48
Inferior occipital/Cuneus	18	58	30	−85	−20
**HEARING > DEAF NATIVE SIGNERS**					
**Left**					
Post-/Precentral gyrus	3/4	73	−58	−21	44
Inferior/Superior parietal lobule	40/7	49	−26	−41	64
**Right**					
Precentral gyrus	4	25	62	−1	12
Insula	13	23	42	−17	16
Postcentral gyrus	2/3	219	30	−33	68

Two of the regions identified as showing greater connectivity with left STG were of special interest, due to their supposed involvement with the semantic/ventral network (BA 45) and phonological/dorsal network (postcentral gyrus). Connectivity from left STG in these two regions was computed for the oral deaf group. Connectivity between the left STG and BA 45 was significantly greater in oral deaf than hearing, *t*_(26)_ = 2.23, *d* = 0.85, *p* = 0.04, but not significantly different than that of native signers, *t*_(26)_ = −0.86, *d* = 0.31, *p* = 0.40. Importantly, oral deaf patterned differently when examining the connectivity between the left STG and left postcentral gyrus. Connectivity in oral deaf was not significant different than that of hearing, *t*_(26)_ = −1.42, *d* = 0.55, *p* = 0.17, but greater than that of deaf native signers, *t*_(26)_ = 3.07, *d* = 1.14, *p* = 0.005.

#### Understanding neuroplastic changes in the semantic and phonological networks

In addition, we took advantage of the different regions within inferior frontal cortex that have been associated with distinct semantic (BA 45/pars triangularis) and phonological/syntactic (BA 44/pars opercularis) processing respectively (Dapretto and Bookheimer, 1999; Hagoort, 2005; Costafreda et al., 2006; Heim et al., 2008). Functional connectivity analyses seeding from these two distinct anatomically defined regions (BA 44, 82 voxels, center of mass: −50, 7, 11; BA 45, 149 voxels, center of mass: −48, 28, 16) were performed to further investigate the differences in the dorsal/phonological versus ventral/semantic networks of reading in the three populations studied. The same procedure as above was followed, first contrasting functional connectivity in deaf native signers vs. hearing readers when seeding from these areas. Then, using the so-defined projection regions, connectivity in that same pathway was computed in oral deaf and pair-wise comparisons were performed against deaf signers and hearing.

***Pars triangularis(BA45)***. Seeding from left BA45, greater connectivity was observed in deaf native signers than in hearing individuals toward the left and right STG (see Table 5 for all areas). Connectivity of the same projection in oral deaf was not significantly different than that of deaf native signers, *t*_(26)_ = −0.13, *d* = 0.05, *p* = 0.90. but greater than that of hearing, *t*_(26)_ = 2.93, *d* = 1.10, *p* = 0.007. These results closely mirror the connectivity analysis reported above when seeding from left STG. In addition, greater connectivity in hearing than deaf signers was found toward the left posterior middle temporal gyrus, an area that has been associated with lexical semantics (Mummery et al., 1999; Gitelman et al., 2005).

**Table 5:** **Regions in which functional connectivity with LIFG pars triangularis (BA 45) differed between hearing and deaf native signers, *p* < 0.05 (corrected)**.

**Label**	***BA***	**No. Voxels**	***X***	***Y***	***Z***
**DEAF NATIVE SIGNERS > HEARING**					
Left superior temporal gyrus	41/42/22	38	−54	−13	8
Right superior temporal gyrus	41/22	26	54	−5	4
**HEARING > DEAF NATIVE SIGNERS**					
Left posterior middle temporal gyrus	39/19	22	−54	−61	16
Left cuneus/Middle occipital gyrus	18	22	−26	−89	24

***Pars Opercularis (BA44)***. Seeding from left BA 44, greater connectivity was found in hearing individuals than in deaf native signers toward the posterior fusiform gyrus (BA 18/19/37). This area has been linked with phonological processing, in particular grapheme to phoneme conversion, in reading (Shaywitz et al., 2002; Dietz et al., 2005; Hillis et al., 2005). Connectivity of the same projection in oral deaf was greater than in deaf native signers, *t*_(26)_ = 2.09, *d* = 0.80, *p* = 0.047, but lesser than in hearing, *t*_(26)_ = −3.17, *d* = 1.24, *p* = 0.004. No areas showed significantly greater connectivity in deaf native signers than hearing (see Table 6).

**Table 6:** **Regions in which functional connectivity with LIFG pars opercularis (BA 44) differed between hearing and deaf native signers, *p* < 0.05 (corrected)**.

**Label**	***BA***	**No. Voxels**	***X***	***Y***	***Z***
**HEARING > DEAF NATIVE SIGNERS**					
**Left**					
Posterior fusiform	18/19/37	57	−46	−65	−16
Posterior middle temporal gyrus	39	19	−50	−65	12
**Right**					
Precuneus	7/31	33	26	−69	32

## Discussion

The neural networks that mediate sentence reading in deaf individuals were investigated in three populations with different language background and depth of hearing loss—that is deaf native signers, oral deaf and hearing controls. The participants in all three groups were closely matched for reading skills as measured by a test of sentence comprehension. They differed markedly, however, (i) in terms of language experience, with deaf native signers relying on ASL but oral deaf and hearing relying on English for communication, and (ii) in terms of hearing loss with all deaf participants being congenitally deaf. Each of the three groups engaged a largely similar network of regions often associated with reading, including areas associated with a ventral reading network (middle temporal gyrus, the LIFG pars triangularis BA 45/47) and areas associated with a dorsal reading network (superior temporal areas, the supramarginal gyrus, the pre- and post-central gyri, and the opercular part of the inferior frontal gyrus, BA44). A striking group difference was noted, however, in the engagement of the superior temporal gyri whereby deaf individuals showed greater recruitment. Interestingly, the strength of the activation was best predicted by severity of dB loss among deaf individuals in the left STG. In addition, left STG displayed greater functional connectivity to a major node in the ventral network, the LIFG (BA45), in deaf individuals, in accordance with greater reliance on semantic processing during reading in this population. Furthermore, we confirmed greater connectivity from the left STG to dorsal, more speech-based areas of the reading network such as the left postcentral gyrus, in hearing and in oral deaf participants as compared to deaf native signers. As these former groups are known to make greater use of English as a mode of communication, this finding is in accordance with the importance of “phonological awareness” and speech-based analysis at large during reading in such populations.

### A common network

As previously reported in the literature, a common network of areas was found to be recruited across all three populations during sentence reading. This network included the typical reading areas in the left perisylvian regions (e.g., middle as well as superior temporal cortex and left inferior frontal cortex) and left inferior temporal cortex, insula and the pre-/postcentral gyrus (Fiebach et al., 2002; Dehaene, 2009), in addition to the right homologs of middle temporal and inferior frontal gyri (Carpenter et al., 1995). This suggests reading comprehension is largely mediated by the same reading network, regardless of sensory or language experience. These results are consistent with the past studies of sentence reading in deaf native signers (Neville et al., 1998; Newman et al., 2002) as well as more recent work using exclusively single word reading (Aparicio et al., 2007; Waters et al., 2007; Corina et al., 2013; Emmorey et al., 2013). These latter studies also indicated recruitment of the VWFA in deaf readers as observed here. However, in the present study, hearing and oral deaf participants displayed greater sentences vs. falsefont activation in the VWFA as compared to deaf native signers. If confirmed, the source of this group difference will have to be further characterized.

### Superior temporal gyrus

Our results demonstrate greater recruitment of the STG bilaterally in deaf signers and in oral deaf as compared to hearing individuals. This suggests a role of auditory deprivation in the reorganization of the STG bilaterally, and is in agreement with the extant literature on cross modal plasticity. Surprisingly, only activation in the left STG was sensitive to the gradation in dB loss, but not that in the right STG, possibly owing to our use of a language task. Indeed, depending on the task used, the literature indicates predominantly right versus left STG reorganization in deaf individuals. The majority of past research showing left STG recruitment in deaf individuals focused on sign language processing (MacSweeney et al., 2002, 2004, 2006; Cardin et al., 2013), and that showing right STG recruitment focused on non-linguistic visual processing (Finney et al., 2001; Fine et al., 2005).

Concerning the role of the STG, the recent study by Cardin et al. is of special interest as it is among the rare studies like ours to include both a deaf signer group and an oral deaf group in addition to a hearing group. They document enhanced activation in the right STG for both deaf signers and oral deaf as compared to hearing, but only greater left STG recruitment in deaf signers and not oral deaf when processing sign language. They conclude that the reorganization of the right STG is driven by auditory deprivation and sign language use and that of the left STG is more driven exclusively by sign language use. On the surface, their results may seem contradictory to our current results, especially regarding the left STG. However, the tasks performed in the Cardin study and previously reported studies documenting left STG reorganization in the deaf were comparing sign language processing to non-linguistic stimuli. Therefore, the sign language stimuli would only activate areas recruited for language processing in deaf signers, but not in oral deaf nor in hearing controls (who are typically non signers). In contrast, in the current study, subjects were reading for comprehension in English, with similar levels of proficiency across groups. Thus, the task was a “language” task for all participants. The current study therefore offers an interpretation of left STG recruitment in the deaf as possibly linked to its functional reorganization for aspects of visually-mediated language processing. This interpretation is consistent with its recruitment only in deaf signers during a sign language task, but in both deaf signers and oral deaf during a reading task.

### Functional connectivity with left STG and its implications for reading

In the absence of sensitive online measures of reading processing like eye-tracking or self-paced reading (Bélanger et al., 2012, 2013), group differences in the functional connectivity with left STG provided some further insight into its functional role in deaf readers. The stronger connectivity from left STG to BA 45 in deaf signers as compared to hearing readers suggests a greater reliance on the ventral reading network and possibly the use of lexical-semantics and conceptual schemas in congenitally deaf signers. This view is also supported by the greater connectivity noted between left STG and the thalamus, as this structure has also been implicated in semantic related language mechanisms (Botez and Barbeau, 1971; Crosson, 1999; Johnson and Ojemann, 2000; Ketteler et al., 2008). For example, the thalamus is sensitive to concreteness (Friederici et al., 2000), and has been implicated in controlled (i.e., not automatic) semantic ambiguity resolution (Ketteler et al., 2008) as well as in regulating and monitoring segments for semantic verification. The thalamus was a consistent area that showed greater functional connectivity with the bilateral STG in deaf native signers compared to hearing. Interestingly, increased functional connectivity from deprived cortex to inferior frontal cortex and thalamus was also reported in blind individuals (Bedny et al., 2011). Thus, cross-modal plasticity may not only lead to greater recruitment of deprived areas (visual for blind and auditory for deaf), but also to new connectivity with the deprived area maintaining strong functional connectivity with the thalamus and inferior frontal areas typically engaged during language processing. A fruitful avenue for future research will be to explore the possibility that the reading network of deaf native signers, including left STG along with its functional connectivity with left IFG (BA 45) and the thalamus, reflect a greater reliance on semantic processing during sentence comprehension.

Interestingly, the same left STG connectivity to LIFG/BA45 was observed in oral deaf as in native signers indicating that early signing is not necessary to foster greater reliance on this pathway. Seeding from the triangular region of the LIFG (BA 45), an area associated with semantic processing and selectively involved in processing the semantic aspects of a sentence (Dapretto and Bookheimer, 1999), we confirmed that there was greater connectivity to bilateral superior temporal gyri in deaf native signers and in oral deaf as compared than hearing. Other works support the view that skilled readers who are profoundly deaf may process written language differently than hearing readers. In particular, enhanced attentional allocation to the parafovea while reading has been documented (Bélanger et al., 2012) with previous work identifying hearing loss, and not language background, as the determinant of a broader window of attention (Dye et al., 2009). These findings have not been explicitly linked with greater reliance on semantic processing in skilled deaf readers, but combined with research suggesting that deaf readers make a lesser use of phonological information when reading, the hypothesis that deaf individuals are better able to integrate upcoming semantic information and process the global gist while reading could be a fruitful direction for future research.

The connectivity from left STG to post-central and from LIFG/BA44 to poster parts of the reading network, areas all identified as part of the dorsal/phonological reading and language networks, provides an additional source of information when it comes to understanding the functional role of the left STG. These dorsal projections were stronger in hearing and in oral deaf readers as compared to deaf signers. Thus, familiarity with speech-based information seems important in strengthening the dorsal reading network, and successful oral training appears to promote the development of such connectivity.

### Conclusions

The current data demonstrates largely overlapping reading networks in deaf individuals, whether native signers or trained orally, as compared to hearing controls. Potentially driven by cross-modal plasticity due to hearing loss, the STG, and thus a large part of auditory cortex, exhibited greater recruitment in deaf individuals irrespective of their language background as compared to hearing. This area on the left also showed a tight link with dB loss level and an enhanced functional connectivity as a result of deafness toward the LIFG (BA 45) and the thalamus, two areas that have been implicated in the processing of semantic information during language processing. Interestingly, connectivity between the left STG and other areas of the dorsal, more speech-based reading network was found to be strengthened in hearing but also oral deaf as compared to native signers. Taken together, this work suggests similar pathways for reading comprehension in deaf and hearing, but possibly differential reliance on these pathways as a function of hearing loss and language skills.

### Conflict of interest statement

The authors declare that the research was conducted in the absence of any commercial or financial relationships that could be construed as a potential conflict of interest.

## Footnotes

^1^One deaf native signer had unaided dB loss of 70 and one oral deaf had unaided dB loss of 63.

^2^Two oral deaf became deaf at age 4.
