# Supplementary material for: Neural networks mediating sentence reading in the deaf
Source: Front Hum Neurosci. 2014 Jun 10;8:394. doi: 10.3389/fnhum.2014.00394 (PMC4050738; doi:10.3389/fnhum.2014.00394)
Supplement: Supplementary file 1 [file DataSheet1.DOCX]

**Supplemental Information**

**1) Speech-based Phonological Knowledge Tasks**

Performance on a battery of tasks tapping different levels of speech–based knowledge was combined to provide an index of speech-based phonological knowledge; the main features of this battery are described in Hirshorn et al. (under review). We give a succinct account below.

***Phonological Tests****.* All tests were designed to be deaf friendly and used black and white pictures ([primarily from Snodgrass & Vanderwart, 1980](#_ENREF_4)), to avoid reading, per se. Before each test, participants were shown each picture and asked to type its name. Feedback was provided and if an error was made, the item was retested at a later time in the set to insure proper naming.

Four tasks were used to derive a comprehensive measure of English phonological knowledge.

(i) *Phoneme odd-man-out task.* Participants had to find the “odd-man-out” among three pictures one of which had a different first-sound (doll/door/belt; for half of the items,) or a different vowel (steak/chain/leaf; for the other half – each half presented in separate blocks). Half of the trials for both first-sound and vowel blocks were more transparent, where spelling knowledge alone would lead to the correct answer. The other half was more opaque such that spelling knowledge alone would load to the wrong answer.

(ii) *Phoneme manipulation task.* Participants viewed two pictures and had to take the onset of the first picture and combine it with the rime of the second picture to make a new real word (which they then typed out). There were an equal number of transparent trials (e.g., the onset of 'mop' + rime of 'rug'= mug) and opaque trials (e.g., the onset of 'king' + rime of 'bowl' = coal).

(iii) *Syllable task.* Participants were presented with an ‘odd man out’ and asked to choose the picture whose name had a different number of syllables than the other two. The odd man could either have more or less syllables than the other two items (e.g., lemon/clock/sheep or glass/table/paper).

(iv) *Speechreading task.* The sentence-level task developed by Mohammed and colleagues was adapted to American English ([Mohammed, Campbell, Macsweeney, Barry, & Coleman, 2006](#_ENREF_2); [Mohammed, MacSweeney, & Campbell, 2003](#_ENREF_3)). Briefly, participants viewed spoken sentences (with no sound) enunciated by an interpreter with native ASL skills and had to decide which of 6 pictures was the best match to the sentence just viewed.

A composite score was calculated by averaging z-scores for all four speech-based knowledge measures: phoneme odd-man-out, phoneme manipulation, syllable task, and speechreading.

**2) Additional fMRI Sentence Task Details**

Half of the sentences that were followed by a mismatching picture, half had an incorrect action (e.g., the waiter was pouring, not spilling the wine) and half had an incorrect object (e.g. the butterfly was sitting on the window, not a flower). Participants did not know if picture probes would appear in order to promote reading for comprehension on all trials. Participants were instructed to decide if the picture matched the sentence they just read or not. Sentences, both followed by a picture and without a picture, were an equal mix of different levels of syntactic complexity (subject/verb/object, conjoined, and passive), but for all analyses reported, they were merged together.

**3) Sentence Processing areas in each group**

Table S1. Hearing: Sentences vs. Falsefont

Table S2. Orally-Trained Deaf: Sentences vs. Falsefont

Table S3. Native Signers: Sentences vs. Falsefont

**4) Functional Connectivity from Right STG**

Seeding from right STG, the set of regions that showed group differences between deaf native signers and hearing mirrored what was seen from seeding from the left STG. The bilateral IFG, insula, and thalamus, as well as the left fusiform showed greater connectivity with deaf native signers than hearing. Conversely, bilateral postcentral gyrus showed greater connectivity in hearing than deaf native signers.

Overall these analyses reveal a richer set of functional connectivity from the STG bilaterally in the deaf as compared to hearing subjects, with in particular a more robust connectivity toward the inferior frontal gyri (BA 45).

Table S4. Regions in which functional connectivity with right STG linearly varied as a function of strength across groups, *p*<.05 (corrected).

**References**

Hirshorn, E. A., Dye, M.W.G., Hauser, P.C., Supalla, T., & Bavelier, D. (under review). Achieving Literacy in profoundly deaf individuals: The role of early language experience.

Mohammed, T., Campbell, R., Macsweeney, M., Barry, F., & Coleman, M. (2006). Speechreading and its association with reading among deaf, hearing and dyslexic individuals. *Clinical linguistics & phonetics, 20*(7-8), 621-630.

Mohammed, T. E., MacSweeney, M., & Campbell, R. (2003). *Developing the TAS: Individual differences in silent speechreading, reading and phonological awareness in deaf and hearing speechreaders*.

Snodgrass, J. G., & Vanderwart, M. (1980). A standardized set of 260 pictures: norms for name agreement, familiarity, and visual complexity. *Journal of Experimental Psychology: Human, Learning and Memory, 6*, 174-205.
